# Temporal trajectories of real-life hospital laboratory data measured during usual care predict clinical outcome in patients with HFpEF in both sexes

**DOI:** 10.1016/j.ijcrp.2026.200720

**Published:** 2026-10-19

**Authors:** Ezgi Hatip Unlu, Thomas F. Kok, Mylène Barry – Loncq de Jong, Pieter F. van den Berg, Navin Suthahar, Robert M.A. van der Boon, Wouter Meijers, Rudolf A. de Boer, Jasper J. Brugts, Eric Boersma, Isabella Kardys

**Affiliations:** Erasmus MC, Cardiovascular Institute, Thorax Centre, Department of Cardiology, Rotterdam, the Netherlands

## Abstract

**Background:**

Previous studies using blood biomarkers for prognostication in HFpEF relied on one or two measurements, which does not fully capture dynamic patterns over time. We investigated serially measured hospital laboratory values in relation to all-cause mortality and explored potential sex differences in these associations.

**Methods:**

We examined a cohort of ambulatory HFpEF patients from the outpatient clinic of Erasmus MC, the Netherlands, between 2017 and 2022. Data on 32 laboratory values, several clinical characteristics, and outcomes were extracted from electronic health records. Associations between time-varying laboratory values and all-cause mortality were analyzed using joint modelling, including interaction terms to assess potential sex differences.

**Results:**

In 515 patients, median (25th–75th percentile) age was 73(61, 81) years, and 288(56%) were female. During a median follow-up of 2.9(1.6, 4.3) years, 153(30%) patients were deceased. Several characteristics and laboratory values differed between sexes at baseline. Serial measurements of 23 laboratory values were significantly associated with all-cause mortality. ALT, total bilirubin, HDL cholesterol, LDL cholesterol, and triglycerides exhibited statistically significant sex differences in these associations, and for some, statistical significance persisted exclusively in either males or females. In five different multivariable models, NT-proBNP maintained statistical significance, alongside several others. Yet, the incremental discriminative abilities of these models remained limited compared to NT-proBNP only, with the highest AUC reaching 0.715.

**Conclusion:**

Sex-based differences for prognostication in HFpEF were observed for serially measured lipid and hepatic laboratory values. Serially measured NT-proBNP was most robust, and while other laboratory values were also independent predictors, their incremental discriminative value was limited.

## Graphical abstract

**Figure:**
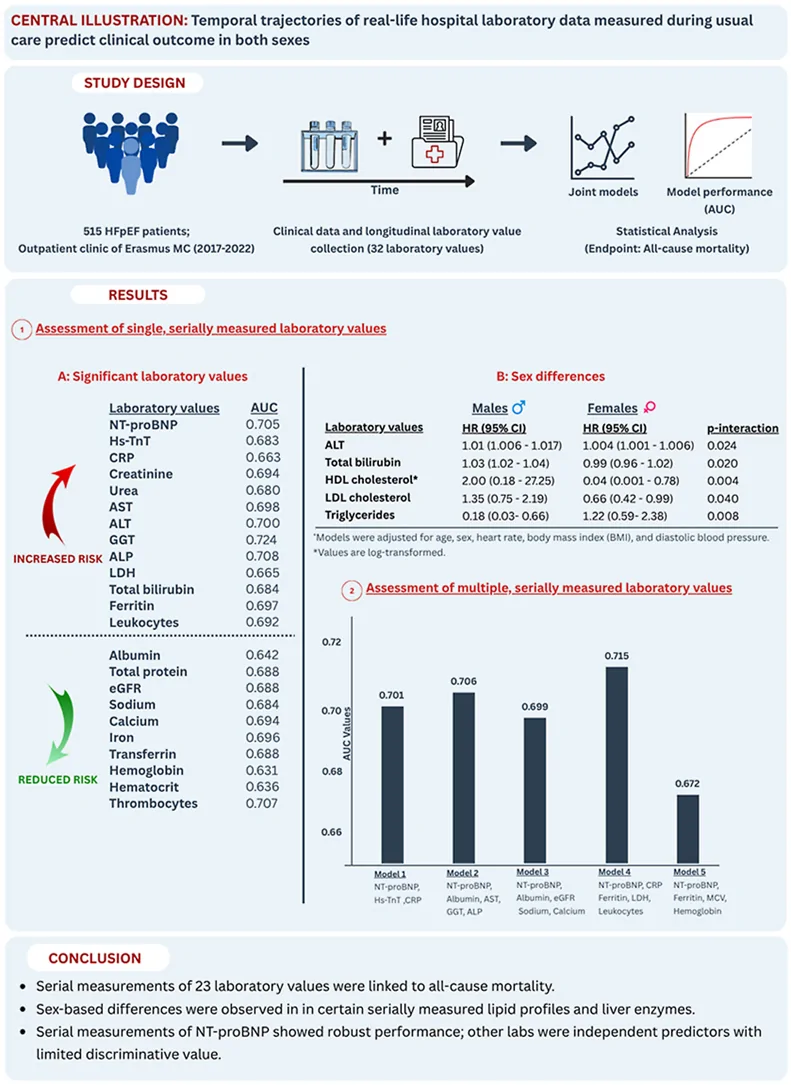

## 1 Introduction

Heart failure with preserved ejection fraction (HFpEF) represents approximately half of all heart failure (HF) cases [1] and is diagnosed multimodally through clinical examination, NTproBNP, echocardiography, and additional diagnostics [2]. Patients are generally older compared to patients with heart failure with reduced ejection fraction (HFrEF), predominantly female [3], and usually have a high prevalence of chronic comorbidities, numerous hospital admissions, and poor survival rates [4], [5].

Prognostication and management of HFpEF remain challenging due to its complex and heterogeneous nature, as well as overlap in symptoms and signs with other conditions [6]. Blood biomarkers offer a potential tool for early detection of disease progression, often before clinical symptoms become apparent [7]. Therefore, routine monitoring of accessible and easily measurable laboratory values could help identify patients at higher risk for adverse outcomes [7], [8].

Several studies have investigated diverse biomarker categories (cardiac, renal, and hepatic) in HFpEF in different research settings, to evaluate their associations with clinical outcomes [9], [10], [11]. However, most of these studies focused primarily on single, ‘baseline’ biomarker measurements in relation to clinical outcome, or on changes in biomarker levels between two time-points. Although a proven association exists between biomarkers and various clinical outcomes, relying on one or two measurements does not fully capture their dynamic patterns over time. Furthermore, most biomarkers were evaluated by non-standard analytical techniques. The specialized assays required in most cases preclude routine use of most of the biomarkers studied. Moreover, blood biomarker levels and associations with outcomes may vary between sexes due to a range of factors, including hormonal, genetic, and physiological variations [12]. Sex differences in biomarkers have been widely described across the spectrum of HF [3], [13], [14], but few studies have examined sex differences in commonly used clinical laboratory values in HFpEF patients [15], [16]. Additionally, these studies have generally not addressed sex differences in longitudinal biomarker trajectories.

Consequently, there is a need to identify temporal patterns in routinely used laboratory values that can enhance risk assessment and prognostication in HFpEF. Previous studies have been limited by homogeneous patient populations and strict study protocols, which may not reflect the diversity seen in real-world cohorts. In contrast, real-life data, derived from a heterogeneous patient population without the selective inclusion/exclusion criteria found in study settings, could provide a more accurate representation of intra-patient disease trajectories, and herewith enable early detection of progression [17]. Moreover, the combination of multiple laboratory values could improve risk assessment [18], [19]. We have recently demonstrated in a real-world study in an HFrEF–HFmEF population that using routine laboratory values facilitates risk stratification without necessitating additional effort [20]. However, prognostic value remains underexplored in real-life data in HFpEF patients.

Therefore, we aimed to investigate the associations of serially measured laboratory values, measured during usual care and recorded in electronic health records, with all-cause mortality in ambulatory patients with HFpEF. We also sought to address the relevance of organ system-specific sets of laboratory values and to explore potential sex differences in these associations.

## 2 Methods

### 2.1 Data acquisition

In this retrospective cohort study, patient data was extracted from the Healthcare Information eXchange (HiX), the electronic health record powered by ChipSoftTM, and implemented in the Erasmus MC, Rotterdam, the Netherlands, in June 2017. We extracted data on all HF patients who had an outpatient visit at the Cardiology department between the implementation of HiX and April 2022. The follow-up period lasted until April 2023, and accordingly, all patients had at least one year of follow-up available.

This study was exempt from the Dutch Medical Research Involving Human Subjects Act (WMO) according to the Institutional Review Board of Erasmus MC, because of the retrospective nature of the study and the use of anonymous data. Approval for patient data acquisition was obtained via a ‘non-WMO’ declaration, and in line with the retrospective and anonymous nature of the study, the need for individual informed consent was waived (MEC-2022-0361).

### 2.2 Selection of HFpEF patients

The presence of HF was ascertained based on in-hospital medical diagnoses, which had been labeled with the 10th revision of the International Classification of Diseases (ICD-10) codes I50.0, I50.1, or I50.9. In the absence of these codes, financial information was used to identify HF patients, as obtained from a system used in the Dutch healthcare sector to streamline the billing process for medical treatments; specifically, ‘Diagnosis Treatment Combination’ (DBC) codes CAR.301 and CAR.302 were applied.

In total, we identified 2778 HF patients based on in-hospital medical diagnoses (Fig. 1). The current definition of the European Society of Cardiology (ESC) requires detailed phenotyping, which is not always feasible in clinical care [2]. Therefore, we used a simplified phenotyping protocol. Herein, HFpEF participants were identified through echocardiography reports containing a quantitative assessment (LVEF ≥50%) or a qualitative assessment (visual estimation; good systolic function (as opposed to mild, moderate, moderate to severe or severe)) at the baseline visit, and biomarkers did not play any role in the selection of patients. We excluded patients <18 years at the time of their first available outpatient visit. This resulted in a final HFpEF study population of n = 515.

**Fig. 1:**
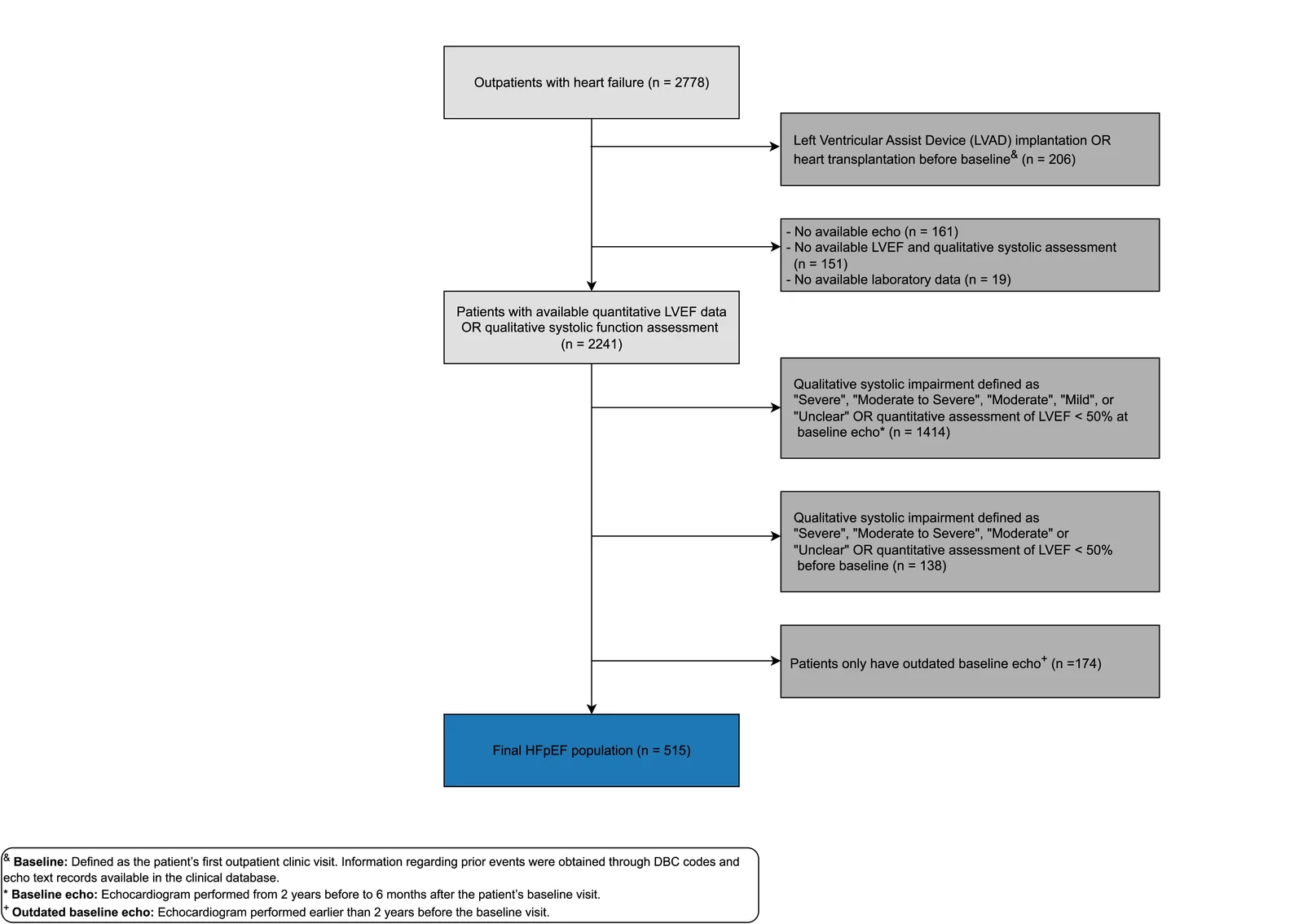
Flow chart of the HFpEF study population, showing patient selection and exclusions.

### 2.3 Data collection and definitions

Following cohort identification, physical characteristics, such as age (at first available outpatient visit), sex, clinical variables (body mass index (BMI), heart rate, diastolic blood pressure (DBP), and medication use, and 32 routinely measured laboratory values were selected out of the available variables. Sex was defined as a biological characteristic (male/female) based on data recorded in the electronic health records. Notably, clinical characteristics used in this investigation were limited to those registered in well-structured fields in the electronic health record, instead of free text.

### 2.4 Endpoints

The study endpoint was all-cause mortality (ACM). Mortality- and censoring dates were obtained through a batch-wise check of the municipality database.

### 2.5 Statistical analyses

Continuous variables are presented as medians and 25th −75th percentiles. Categorical variables are presented as numbers and percentages. Variable distributions were examined using histograms and QQ-plots and tested for normality through the Anderson-Darling test. If the distribution was skewed, the variable was base e log transformed. Baseline characteristics were compared between sexes using Mann-Whitney *U* test for continuous variables, and $\chi^{2}$ test or Fisher's exact test for categorical variables. Survival time was defined as the time difference between the date of a patient's first registered outpatient visit in or after June 2017, and the censoring date or ACM date.

We explored associations of serially measured laboratory values with ACM using joint models [21]. Details regarding joint modeling and model selection can be found in the Supplementary materials. We performed both single-laboratory value and multiple-laboratory value analyses, and we included all participants in the multivariable analyses, regardless of whether they had specific biomarkers measured or not, to benefit from their contribution to the survival part of the data. To examine the potential influence of measurement frequency, we compared models adjusted for baseline clinical variables alone with models additionally adjusted for the number of repeated measurements as a time-varying covariate. Deviance Information Criterion (DIC) was used to compare models. Sex differences were analyzed by adding interaction terms between sex and longitudinal laboratory value trajectories to the models. As a sensitivity analysis, for statistically significant interactions, p-interaction was also tested while adjusting for time-varying measurements.

We used five multiple-laboratory value joint models to explore the association of various combinations of values with ACM. We used clinical knowledge, results of single laboratory-value models, and correlation plots (Supplemental Fig. 1) for the selection process. All models were adjusted for clinical variables (age, sex, BMI, heart rate, and DBP) at baseline. Details of the selection and evaluation of models can be found in the Supplementary materials.

Performance assessments of both single and multivariable joint models were based on the AUC derived from the time-dependent receiver operating characteristic (ROC) curve [21], which used the longitudinal data available during the first two years of follow-up to predict outcomes in the subsequent two years. Model performance was evaluated using the Brier score, Integrated Calibration Index (ICI), and calibration plots, which are commonly used to assess predictive performance in dynamic prediction and joint-model settings [21]. For both the Brier score and ICI, lower values indicate better performance. Additionally, landmark analyses were performed at six months and one year to evaluate model performance using information available up to each landmark time to predict outcomes in the remaining years until year four. To evaluate incremental value of multiple-laboratory value models compared to the ‘single’ NT-proBNP model, confidence intervals for the AUCs of these models were obtained by using bootstrap resampling. Differences between AUCs were assessed using a Wald z-test. Statistical significance was evaluated using the resulting z-statistics and corresponding two sided p-values.

Hazard ratios (HRs), derived from joint models, represent the relative risk per unit increase or log increase, depending on whether the biomarker was log e transformed beforehand, at any point in time during follow-up, between two participants. Missing values were imputed using multiple imputation for only baseline heart rate, DBP, and BMI.

A two-tailed p-value < 0.05 was considered statistically significant for differences in baseline characteristics. The joint models we used are Bayesian models, in which partial pooling acts as an automatic correction for multiple comparisons [21]. Statistical analysis was done in R (R-4.5.1) using the packages “nlme”, “JMBayes2”, “survival”,”JointAI”, “tidycmprsk” and “ggsurvfit”.

## 3 Results

The study population (n = 515) had a median (25th–75th percentile) age of 73 (61, 81) years, with 288 (56%) females. Median (25th–75th percentile) values of baseline laboratory values and medication use are presented in Table 1. Baseline comparisons between male and female patients showed that females were more likely to be older, to have lower body weight, and lower median levels of creatinine, urea, ALT, GGT, ferritin, and hemoglobin (Table 1). Conversely, males had a lower median level of thrombocytes and leukocytes. Medication use was similar between sexes except for statins, which were prescribed less frequently (36%) in females compared to males (47%). During a median follow-up of 2.9 (1.6, 4.3) years, a total of 153 (30%) patients were deceased. Females had a median (25th–75th percentile) of 3 (1.8, 4.2) years of follow-up, and 81 (28%) of them were deceased. Males had a median follow-up of 2.7 (1.4, 4.4) years, and 72 (32%) of them were deceased. There were no statistically significant sex differences in follow-up times or ACM.

**Table 1:** Overview of baseline demographic data, biomarker levels, and medication use.

Variables	All (n = 515)	Males (n = 227)	Females (n = 288)	p-value
Age(years)	73(61,81)	70(61,80)	74(62,82)	**0.030**
Weight(kg)	77(67,90)	82(72,95)	73(63,84)	**<0.001**
BMI(kg/m2)	27(23,31)	27(23,31)	26(24,31)	0.991
Heart rate(bpm)	75(66,86)	74(66,84)	75(65,88)	0.672
SBP(mmHg)	135(121,151)	133(119,147)	138(123,154)	0.058
DBP(mmHg)	74(64,82)	75(65,82)	74(64,83)	0.853
NT-proBNP(pmol/l)	75(34,193)	78(26,202)	73(37,167)	0.904
Hs-TnT(ng/l)	23(16,48)	24(16,48)	22(17,52)	0.882
CRP(mg/l)	4(1,9)	4(2,18)	3(1,7)	0.075
CK(U/l)	90(67,155)	91(67,184)	86(68,125)	0.655
CK-MB mass(μg/l)	4(2,7)	3(2,7)	4(2,7)	0.934
Albumin(g/l)	39(35,43)	38(35,42)	39(36,43)	0.382
Total protein(g/l)	69(63,76)	70(65,75)	69(63,79)	0.750
Creatinine(umol/l)	101(76,130)	113(94,148)	91(70,117)	**<0.001**
Urea(mmol/l)	8(6,12)	9(6,13)	7(6,11)	**0.010**
CKD-EPI eGFR(ml/min)	54(41,72)	52(41,70)	54(41,76)	0.347
Sodium(mmol/l)	140(138,142)	140(138,142)	141(139,142)	0.158
Potassium(mmol/l)	4.3(4.0,4.7)	4.4(4.1,4.7)	4.3(4.0,4.6)	0.058
Calcium(mg/dl)	9.4(9.0,9.8)	9.5(9.0,9.8)	9.4(9.0,9.8)	0.571
AST(U/l)	27(20,36)	28(21,37)	26(20,36)	0.384
ALT(U/l)	23(15,33)	26(17,41)	20(14,28)	**0.021**
GGT(U/l)	37(21,93)	52(27,154)	29(19,44)	**<0.001**
ALP(U/l)	85(66,113)	90(64,127)	83(67,105)	0.305
LDH(U/L)	236(180,289)	226(175,275)	246(190,293)	0.300
Total bilirubin(μmol/l)	8(6,12)	8(6,13.375)	8(5,11)	0.473
Total cholesterol(mmol/l)	4.5(3.7,5.5)	4.4(3.6,5.5)	4.9(3.8,5.6)	0.500
HDL cholesterol(mmol/l)	1.6(1.2,2.2)	1.3(0.9,2.3)	1.7(1.4,2.2)	0.127
LDL cholesterol(mmol/l)	2.9(2.2,3.7)	2.8(2.0,3.5)	2.9(2.3,4.0)	0.189
Triglycerides(mmol/l)	1.9(1.1,2.4)	2.0(1.0,2.7)	1.8(1.3,2.1)	0.492
Ferritin(μg/l)	182(57,475)	277.5(92,516)	95(42,340)	**0.031**
Iron(μmol/l)	13(6.7,18.4)	14.1(11.1,19.6)	8.0(5.4,15.8)	0.066
Transferrin(g/l)	2.5(2.1,2.8)	2.5(2.1,2.7)	2.4(2.1,2.8)	0.84
Haemoglobin(mmol/l)	7.7(6.9,8.3)	7.8(7,8.6)	7.6(6.9,8.1)	**0.023**
Haematocrit(%)	37(33,41)	37(34,42)	37(33,41)	0.354
MCV(fL)	92(88,97)	92(89,98)	91(88,96)	0.196
Leukocytes(109/l)	7.2(5.7,8.9)	6.8(5.4,8.5)	7.6(6.1,9.6)	**0.021**
Thrombocytes(109/l)	209(159,268)	193(145,246)	240(184,285)	**<0.001**
HbA1c(mmol/mol)	45(37,57)	45(37,54)	53(38,65)	0.657

**Medication**^a^	**All (n = 375)**	**Males (n=164)**	**Females (n=211)**	**p-value**

Beta-blockers	207(55%)	91(55%)	116(55%)	1
ACE-inhibitors	130(35%)	62(38%)	68(32%)	0.309
ARBs	98(26%)	36(22%)	62(29%)	0.132
Loop diuretics	227(61%)	103(63%)	124(59%)	0.492
Potassium sparing diuretics	110(29%)	51(31%)	59(28%)	0.584
Statins	153(41%)	77(47%)	76(36%)	**0.042**
Xanthine-oxidase inhibitor	21(6%)	11(7%)	10(5%)	0.551

Values are presented as median (25th and 75th percentiles) for continuous variables and as number (percentage%) for categorical variables. **BMI:** Body mass index; **SBP:** Systolic blood pressure; **DBP:** Diastolic blood pressure; **NT-proBNP:** N-terminal prohormone of brain natriuretic peptide; **Hs-TnT:** High-sensitivity troponin; **CRP:** C reactive protein; **CK:** Creatine kinase; **CK-MB mass:** Creatine kinase – MB mass; **CKD-EPI eGFR:** Glomerular Filtration Rate obtained from CKD-EPI equations; **AST:** Aspartate aminotransferase; **ALT:** Alanine aminotransferase; **GGT:** Gamma-glutamyl transferase; **ALP:** Alkaline phosphatase; **LDH:** Lactase dehydrogenase; **HDL cholesterol:** High-density lipoprotein; **LDL cholesterol:** Low-density lipoprotein; **MCV:** Mean corpuscular volume; **ACE:** Angiotensin-converting-enzyme; **ARB:** Angiotensin receptor blockers.

a Medication data at baseline was available for 375 patients. Only four patients were prescribed SGLT-2 inhibitors.

### 3.1 Longitudinal assessment of individual laboratory values

#### 3.1.1 Total population

Longitudinal characteristics of the dataset and comparison of median numbers of laboratory measurements between men and women are detailed in Table S1. Among the 32 laboratory values with longitudinal trajectories that were assessed, 23 were significantly associated with ACM after adjustment for clinical variables (Fig. 2). Higher levels of NT-proBNP, Hs-TnT, CRP, creatinine, urea, AST, ALT, GGT, ALP, LDH, total bilirubin, ferritin, and leukocytes were each significantly associated with an increased hazard of the outcome. Moreover, several laboratory values were significantly associated with reduced hazards, including albumin, total protein, eGFR, sodium, calcium, iron, transferrin, hemoglobin, hematocrit, and thrombocytes. Detailed HR estimates, including models adjusted only for age and sex, are provided in Table S2. To examine the potential influence of measurement frequency, we compared models adjusted for baseline clinical variables alone, with models additionally adjusted for the number of repeated measurements as a time-varying covariate. Results are presented in Table S3. There was no major change in AUCs, HRs, or their statistical significance, except for eGFR.

**Fig. 2:**
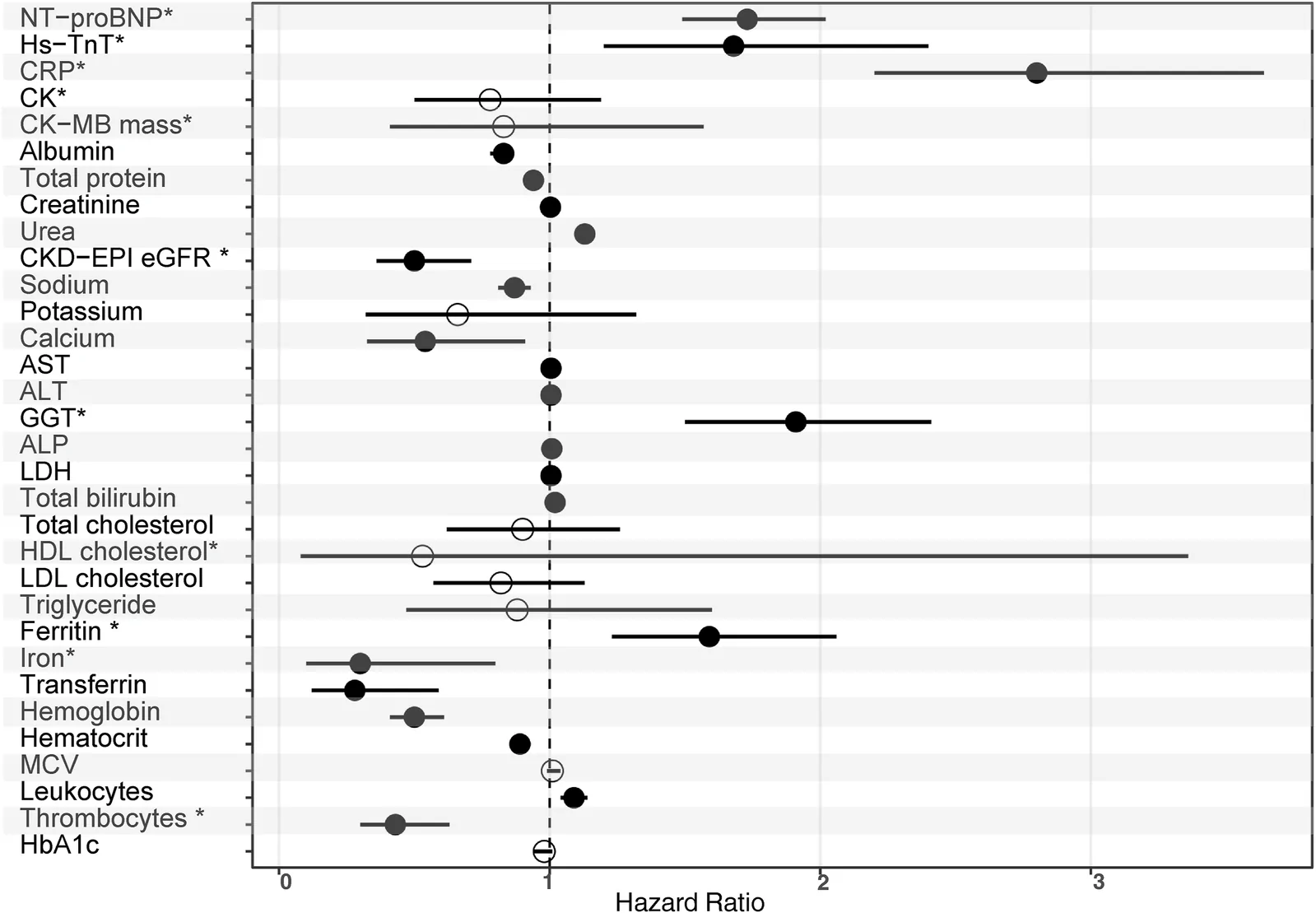
Hazard ratios for single biomarker joint models for ACM. This figure displays the hazard ratios (HRs) and 95% confidence intervals for each biomarker analyzed in univariate joint models. Asterisk (*) indicates variables that are log-transformed before modelling. Filled squares represent statistically significant associations (p < 0.05), while open squares denote non-significant (p ≥ 0.05) results. Models were adjusted for age, sex, heart rate, body mass index (BMI), and diastolic blood pressure.

#### 3.1.2 Sex differences

Among all laboratory values, ALT, total bilirubin, HDL cholesterol, LDL cholesterol and triglycerides exhibited significant sex-specific differences in HRs after adjusting for clinical variables (Fig. 3), with men exhibiting larger HRs for each except triglycerides. Additionally, only in females, elevated total protein, HDL cholesterol, LDL cholesterol, iron, and transferrin levels were linked to lower risk, whereas elevated leukocyte levels were linked to increased risk. Moreover, exclusively in males, elevated triglyceride levels were linked to a lower risk, while higher cardiac troponin T and total bilirubin levels were linked to a higher risk. The remaining laboratory values showed no significant differences in HRs between males and females. Detailed HR estimates and p-values for interaction terms are provided in Table S4 (age adjusted) and Table S5 (adjusted for clinical variables).

**Fig. 3:**
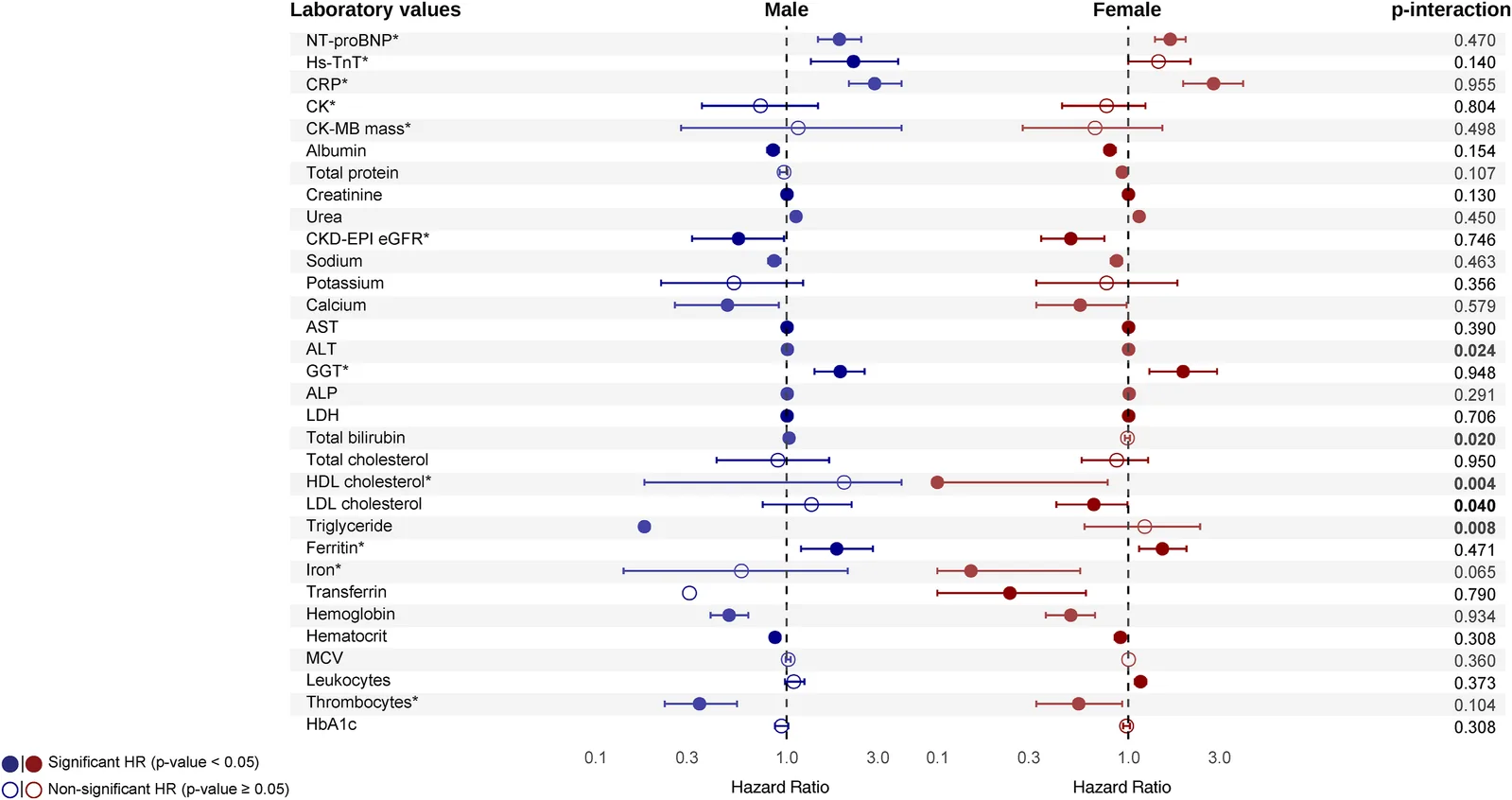
Hazard ratios (HRs) for single biomarker joint models for ACM, in men and women. Asterisk (*) indicates variables that are log-transformed before modelling. Filled symbols indicate statistically significant HR (p < 0.05), while open symbols denote non-significant HR (p ≥ 0.05). p-interaction shows whether HR varies by sex. All models were adjusted for age, sex, heart rate, body mass index (BMI), and diastolic blood pressure.

After additionally adjusting HRs of laboratory values with significant interaction terms for sex, for frequency-of-measurement, the results did not change materially. Results are presented in a footnote in Table S5.

### 3.2 Longitudinal assessment of multiple laboratory values

We examined the longitudinal trajectories of multiple laboratory values in relation to ACM simultaneously, adjusting for clinical variables, using multiple-laboratory value models (Table 2). In each model, NT-proBNP consistently showed a significant association with ACM. Among the other values, CRP, albumin, AST, GGT, eGFR, and hemoglobin remained significant. An age-adjusted comparison of multiple laboratory values is presented in Table S6.

**Table 2:** Longitudinal assessment of multiple biomarkers: multivariable models.

Model	Adjusted for clinical variables			
	Variables	HR (95%CI)	P-value	AUC
Model 1	NT-proBNP^a^	1.38 (1.08-1.80)	0.010	0.701
	Hs-TnT^a^	1.07 (0.63-1.79)	0.797	
	CRP^a^	2.42 (1.87-3.20)	<0.001	
Model 2	NT-proBNP^a^	1.67 (1.38 - 2.04)	<0.001	0.706
	Albumin	0.86 (0.81 - 0.90)	<0.001	
	AST	1.003 (1.0001 - 1.005)	0.049	
	GGT^a^	1.97 (1.37 - 2.85)	<0.001	
	ALP	0.997 (0.99 - 1.003)	0.438	
Model 3	NT-proBNP^a^	2.00 (1.53 - 2.72)	<0.001	0.699
	CKD-EPI eGFR	2.39 (1.31 - 4.71)	0.003	
	Albumin	0.81 (0.75 - 0.86)	<0.001	
	Sodium	0.96 (0.88 - 1.07)	0.446	
	Calcium	1.56 (0.86 – 2.99)	0.147	
Model 4	NT-proBNP^a^	1.31 (1.07 - 1.62)	0.008	0.715
	CRP^a^	2.24 (1.67 – 3.05)	<0.001	
	Ferritin^a^	1.06 (0.73 - 1.53)	0.750	
	LDH	1.004 (1.002 - 1.006)	<0.001	
	Leukocytes	1.02 (0.96 - 1.07)	0.455	
Model 5	NT-proBNP^a^	1.48 (1.21-1.83)	<0.001	0.672
	Ferritin^a^	1.11 (0.79-1.58)	0.527	
	Hemoglobin	0.63 (0.50-0.78)	<0.001	
	MCV	1.005 (0.98-1.03)	0.716	

a Values are log-transformed. **NT-proBNP:** N-terminal prohormone of brain natriuretic peptide; **Hs-TnT:** High-sensitivity troponin; **CRP:** C reactive protein; **CKD-EPI eGFR:** Glomerular Filtration Rate obtained from CKD-EPI equations; **AST:** Aspartate aminotransferase; **ALT:** Alanine aminotransferase; **GGT**: Gamma-glutamyl transferase; **LDH:** Lactase dehydrogenase This table displays the hazard ratios (HRs) and 95% credible intervals for each biomarker analyzed in multivariate joint models. Models were adjusted for age, sex, heart rate, body mass index (BMI), and diastolic blood pressure.

### 3.3 Discriminative ability of the models

To evaluate the discriminatory ability of each (single or multiple laboratory value) model with time-varying ROC curves, information from data collected up to year 2 was used to predict outcomes during consecutive 2-year periods. Fig. 4 presents the performance of both single- and multiple-laboratory value models with adjusted clinical variables. Notably, in the figure, we present only the set of single-laboratory values used in multiple-biomarker models. All single-laboratory value AUCs ranged from 0.631 to 0.724, with the highest observed for GGT (AUC: 0.724), ALP (AUC: 0.708), NT-proBNP (AUC: 0.705), and MCV (AUC: 0.704). Among the multiple-value models, Model 4 (NT-proBNP, CRP, ferritin, LDH, and leukocytes) achieved the highest AUC at 0.715, followed by Models 2, 1, 3 and 5 in descending order of performance. Detailed model performance for other laboratory values and age-adjusted performance for all models is provided in Supplementary Table S2 and Table S6. Landmark analysis for one year, six months and two years (main analysis), including ICI and Brier scores for both single and multiple-laboratory value models, are presented in Table S7. Overall, results showed that both ICI and Brier scores were generally close to zero among all three landmark times, indicating low calibration error and good overall agreement between predicted probabilities and observed outcomes. Calibration plots for both single and multiple-laboratory value models are presented in Table S8. Overall, visual assessment suggested broadly acceptable calibration across all (single or multiple laboratory value) models.

**Fig. 4:**
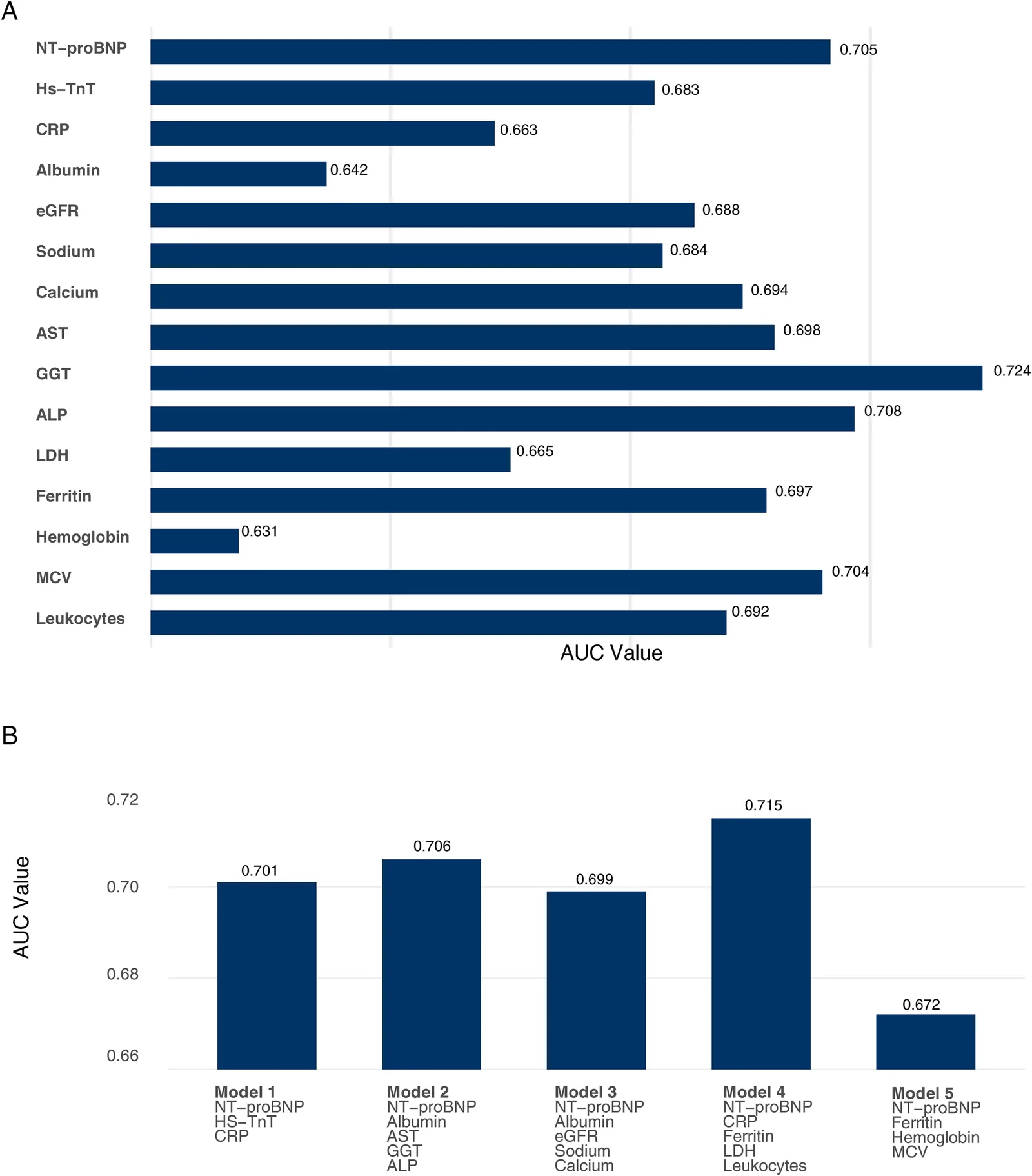
Predictive performance of models based on single and multiple biomarkers **(A)** Single biomarker (only integrated within multiple-biomarker models) predictive ability, expressed as AUC (area under the curve) values, shown with horizontal bars. **(B)** Predictive ability of multiple-biomarker models, shown with vertical bars representing different models (Model 1–5) and their corresponding AUC values. All models were adjusted for age, sex, heart rate, body mass index (BMI), and diastolic blood pressure. For AUC calculation, data up to the 2-year follow-up were used to predict consecutive 2 years.

Discriminative ability of the model containing NT-proBNP alone was compared with each of the five multiple-laboratory value models. None of the multivariable models demonstrated a statistically significant improvement in discrimination compared with NT-proBNP alone (Supplementary Table S9).

## 4 Discussion

In this study, we explored the value of routinely and serially measured real-world hospital laboratory analytes for prognostication in ambulant patients with HFpEF and assessed potential sex differences. Several laboratory values showed significant differences in baseline levels between males and females. In a longitudinal analysis, 23 single-laboratory values were associated with ACM, while certain hepatic and lipid profile values demonstrated sex-specific differences in their associations. Notably, NT-proBNP remained significant across all multiple-laboratory value models, along with a subset of additional laboratory values. However, the discriminative abilities of single and multiple-laboratory value models remained limited. Overall, the inclusion of other laboratory values provided no substantial improvement in predictive performance over NT-proBNP.

This study contributes to the limited evidence on the associations of longitudinal trajectories of laboratory analytes with all-cause mortality in HFpEF, with a particular focus on sex-related differences. Importantly, this real-world HFpEF population, without exclusions, reflects routine clinical practice, in which clinicians interpret laboratory values in the context of prior measurements. However, caution is warranted when evaluating these findings. These laboratory values are typically measured due to clinical indications based on clinician judgment or knowledge, and their use in decision-making may influence patient outcomes. Nonetheless, these real-world data highlight opportunities for prognostication, risk stratification, and optimizing the timing of treatment.

### 4.1 Longitudinal trajectory of biomarkers

In HFpEF, circulating biomarkers can reflect underlying pathophysiological processes, and routinely measured blood biomarkers are representative of these mechanisms [6], [10], [22]. Most prior investigations and meta-analysis assessed prognostic value of one or multiple biomarkers, each demonstrating significant associations with different clinical outcomes [9], [10], [11], but these measurements were most often obtained at the time of HF onset or at a single cross-sectional point in the disease course, while serial measurements were only occasionally considered. A single measurement, however, may not adequately capture a biomarker's dynamic, temporal trajectory. Serial measurement of biomarkers provides incremental value for prognostication. We have demonstrated this previously for HFrEF in study settings [23], [24]. Recently, Lu et al. [8] showed the value of serially measured biomarkers for HFpEF patients in the FINEARTS-HF trial, that they explored the relationship between NT-proBNP and eGFR trajectories and adverse clinical outcomes. Clinically meaningful changes in readily available biomarkers were observed in the months leading up to a clinical event. Our findings are consistent with the FINEARTS-HF trial, which, however, evaluated a limited number of biomarkers and adhered to strict study protocols. Our study extends these findings by incorporating a broader panel of biomarkers and real-world data, derived from a clinical setting with a heterogeneous patient population, with variable intervals between blood sampling, reflecting the differential need for clinical follow-up. This also demonstrates that utilizing data available from electronic health records may improve prognostic strategies, also in a cost-effective manner.

### 4.2 Longitudinal trajectory of biomarkers: sex differences

Biological sex can influence plasma concentrations of blood-based biomarkers and may also modify their impact on disease outcomes [12], [13], [25]. Most studies have focused on sex differences in biomarker levels, but the interaction between sex and biomarker-associated risk remains less explored [12]. Cardiac peptides, particularly natriuretic peptides (NPs), are well-established in heart failure, with levels affected by age, sex, and BMI [26]. However, previous studies showed inconsistent sex-specific findings regarding to biomarkers that previous extensively studied in the context of HF (including NT-proBNP, cardiac troponin T and CRP) and outcomes in HFpEF [15], [16]. Additionally, they were limited to single measurements. In our study, even though higher levels of NT-proBNP, troponin, and CRP were associated with increased risk of ACM, no sex-specific differences were observed after adjustment for clinical factors. These findings suggest that although single time-point biomarker levels may be variable between sexes, serial measurements offer a reliable means for prognostication independent of sex.

For other routinely measured blood-biomarkers, evidence on sex-specific differences in HFpEF patients is limited. In our analysis, we identified several sex-specific differences in the associations between certain hepatic and lipid profile values and ACM. These differences may be partly explained by physiological factors such as muscle mass and sex hormones [12], [27]. Notably, elevated total bilirubin levels have been linked to adverse outcomes in heart failure in RCT settings [11], but sex differences have often been overlooked. In our study, elevated total bilirubin was associated with a higher risk primarily in males, but this relationship was not observed in females. This difference may stem from estrogen, which influences bilirubin metabolism among various pathways [28]. However, we lack data on hormonal status. Moreover, a recent meta-analysis found that higher lipid parameters were associated with lower mortality in heart failure patients [29], which aligns with our findings, except for total cholesterol. Additionally, we observed sex differences in lipid profiles: higher HDL and LDL cholesterol levels were protective only in females, and higher triglyceride levels were protective only in males. These findings should be interpreted cautiously however, due to potential effects of statin use,which has previously been shown to be associated with lower all-cause mortality in HFpEF patients [30]. In our study, baseline statin prescription rate was different between sexes. Additionally, lipid measurements (total cholesterol, LDL cholesterol, HDL cholesterol, and triglycerides) were available for only approximately one-third to one-half of the study population, which resulted in limited statistical power. Furthermore, men were more likely to have LDL cholesterol measurements available. Therefore, differences in lipid-related findings should be interpreted with caution, considering these limitations. Overall, our results align with the notion that there are sex-related differences in biomarker levels, whether natural or disease-related, as well as in the association of biomarkers with prognosis.

### 4.3 Multiple biomarker models

Accurate risk prediction of clinical outcomes could provide guidance for risk stratification and personalized care. Therefore, a comprehensive multi-marker approach may be beneficial to characterize specific HFpEF phenotypes [22]. Previous studies have shown the value of various biomarkers alongside clinical variables to predict various endpoints [18], [19]. In our study, we aimed to demonstrate the intrinsic value of laboratory value combinations rather than to develop a prognostic model. Therefore, we constructed models (established HF markers, hepatic, renal, inflammatory, and anemic) representing each organ system for which laboratory values are readily available. We observed that NT-proBNP maintained significance across all models, alongside albumin, eGFR, AST, GGT, LDH, and CRP, which also remained significant. Other laboratory values lost significance, suggesting that these consistently significant biomarkers may have particular utility in future system-specific prognostic models.

### 4.4 Discriminative ability of models

All single and multiple-laboratory value models showed fair discriminative ability, but multiple-laboratory value models that included biomarkers alongside NT-proBNP did not show better discriminative ability in this population. It should be noted that, as previously demonstrated, testing for improvement in prediction performance is actually redundant if a variable has already been shown to be an independent risk factor, and that standard testing procedures, such as AUCs, are very conservative and thus insensitive to improvements in prediction performance. Nevertheless, to provide an impression of discriminative ability, we presented the AUCs [31]. Notably, we had varying numbers of repeated measurements for each laboratory value in our study. Considering the limited cohort size, the relatively small number of available data points may have limited the reliability of the performance assessments.

### 4.5 Strengths and limitations

An important consideration that must be taken into account when interpreting the results is the nature of our data. This study was conducted in a real-world cohort of ambulatory HFpEF patients at a tertiary hospital, using electronic health record data. Accordingly, laboratory values were measured as part of routine care rather than on a fixed schedule. Thus, these measurements were based on clinician decisions, which, on their part, were based on the patients’ clinical condition, which could have led to bias. Accordingly, healthier patients may have had fewer measurements, leading to non-random missing data. Another consideration is the primary endpoint of ACM. Given that HFpEF has a high prevalence of comorbidities, it is possible that non-cardiac deaths contributed substantially. Consequently, observed biomarker changes may reflect correlations with overall outcomes rather than direct effects on heart failure.

Our study has several strengths. This is the first study that relates serially measured blood analytes to clinical outcomes in a HFpEF population. Furthermore, various pathophysiological pathways were explored for the first time in this population by using multiple-laboratory value models and longitudinal trajectories. Also, the study shows that laboratory values carry prognostic value in a real-world cohort using readily available healthcare records, compared to trial setups.

Some limitations should also be acknowledged. First, data were collected retrospectively during routine outpatient visits, resulting in variability in the availability and frequency of measurements between patients. As patients with more severe disease may undergo more frequent laboratory testing, this could introduce confounding and influence the observed associations between biomarkers and outcomes. To address potential bias related to measurement frequency, we incorporated the number of measurements into our models as a sensitivity analysis; the results remained largely unchanged, suggesting that the observed associations were not substantially affected by differences in measurement frequency. Second, we ascertained HFpEF using a pragmatic definition based on EF, rather than guideline-recommended scores. Third, availability of clinical characteristics and type of study endpoints were restricted by the limited possibilities in our electronic health record system, which only enabled extraction of information from well-structured fields, and not free text. Additionally, we used of all-cause mortality as the outcome, which may may attenuate associations with outcome for predominantly cardiac-specific laboratory values, and may amplify associations for less cardiac-specific laboratory values. Moreover, laboratory values and outcomes were reported by biological sex and we did not specifically analyze differences based on gender identity. Also, data on females’ menopausal status, which may influence biomarkers [32], were not available. Finally, the findings need confirmation in independent cohorts.

## 5 Conclusion

This study shows that real-life hospital laboratory data can be used to predict ACM in an ambulatory HFpEF population. Some laboratory values that were previously extensively studied in the context of HF (including NT-proBNP, cardiac troponin T and CRP) did not exhibit sex differences in associations, whereas several others did. Finally, although several laboratory values demonstrated independent associations with outcome, our analyses revealed no added discriminative ability compared to NT-proBNP alone.

Altogether, our study supports serially measured NT-proBNP as the most robust laboratory value for risk prediction in HFpEF patients and suggests that while other values are also independent predictors, their incremental value is limited, a finding that warrants validation in larger, independent cohorts.

## CRediT authorship contribution statement

**Ezgi Hatip Unlu:** Writing – review & editing, Writing – original draft, Visualization, Methodology, Investigation, Formal analysis, Data curation, Conceptualization. **Thomas F. Kok:** Writing – review & editing, Writing – original draft, Visualization, Methodology, Formal analysis, Data curation, Conceptualization. **Mylène Barry – Loncq de Jong:** Writing – review & editing, Writing – original draft, Investigation. **Pieter F. van den Berg:** Writing – review & editing. **Navin Suthahar:** Writing – review & editing. **Robert M.A. van der Boon:** Writing – review & editing. **Wouter Meijers:** Writing – review & editing. **Rudolf A. de Boer:** Writing – review & editing. **Jasper J. Brugts:** Writing – review & editing. **Eric Boersma:** Writing – review & editing. **Isabella Kardys:** Writing – review & editing, Writing – original draft, Visualization, Supervision, Project administration, Methodology, Investigation, Funding acquisition, Conceptualization.

## Funding

EHU is funded by Dutch 10.13039/100002129Heart Foundation (Grant number: 2020-B004) and 10.13039/501100018722Jaap Schouten Foundation.
