# Supplementary material for: Temporal trajectories of real-life hospital laboratory data measured during usual care predict clinical outcome in patients with HFpEF in both sexes
Source: Int J Cardiol Cardiovasc Risk Prev. 2026 Oct 19;31:200720. doi: 10.1016/j.ijcrp.2026.200720 (PMC13760079; doi:10.1016/j.ijcrp.2026.200720)
Supplement: Supplementary file 1 [file mmc1.docx]

**Temporal trajectories of real-life hospital laboratory data during usual care predict clinical outcome in patients with HFpEF in both sexes: SUPPLEMENT**

Ezgi Hatip Unlu^1^,* Thomas F. Kok^1^,* Mylène Barry – Loncq de Jong^1^, Pieter F. van den Berg^1^, Navin Suthahar^1^, Robert MA. van der Boon^1^, Wouter Meijers^1^, Rudolf A. de Boer^1^, Jasper J. Brugts^1^, Eric Boersma^1^, Isabella Kardys^1^

*Authors contributed equally.

^1^Erasmus MC, Cardiovascular Institute, Thorax Centre, Department of Cardiology, Rotterdam, The Netherlands.

**Statistical analysis**

We explored associations of serially measured laboratory values with all-cause mortality using joint models. Joint models combine linear mixed effects models for longitudinal data, with relative risk models for time-to-event data. We fitted linear mixed-effects models with time (measured in days) as predictors, which were included as both a fixed effect and a random effect. The fixed effect of time was modeled using either natural splines with 2 internal knots or a linear term, and random effects for time were specified as random intercepts, with time modeled either as a linear term or using natural splines with 2 internal knots, depending on model fit (lowest DIC (Deviance Information Criterion), WAIC (Watanabe-Akaike Information Criterion) or LPML (Log Pseudo Marginal Likelihood)) and convergence (Rhat < 1.1). When model fit was broadly comparable, model convergence was prioritized in selecting the final model.

We included all participants; regardless of whether they had the biomarker measured or not, to benefit from their contribution to the survival part of the data. A joint model addresses missing data by capturing the full covariance structure among variables, which allows the model to borrow information across them.

During multiple joint model selection to minimize multicollinearity, only covariates with pairwise correlations |r| < 0.7 were included together in the same model (e.g. eGFR instead of creatinine or urea; ferritin instead of transferrin). The five models were defined as follows: model 1: markers that were previously extensively studied in the context of HF (serial NT-proBNP, High-sensitivity troponin (Hs-TnT), and C reactive protein (CRP)); model 2: NT-proBNP plus liver-related biomarkers (albumin, aspartate aminotransferase (AST), Alkaline phosphatase (ALP), and Gamma- glutamyl transferase (GGT)); model 3: NT-proBNP plus kidney related biomarkers (albumin, eGFR, sodium, calcium); model 4: NT-proBNP plus inflammatory biomarkers (CRP, ferritin, lactase dehydrogenase (LDH) and leukocytes); model 5: NT-proBNP plus anemia-related biomarkers (ferritin, hemoglobin, MCV). The linear mixed-effects part of multiple-laboratory value models were considered based on model fit and convergence. Two models were evaluated: one adjusted for age and sex and another additionally adjusted for clinical variables. The linear mixed-effects model component was kept consistent across models. For the clinical-variable-adjusted models, a simplified model specification (linear term, only random intercept) was used to ensure convergence and model fit in the clinical variable adjusted model, whereas splines (both in fixed and random parts) were used in the age and sex adjusted models to provide adequate convergence (R-hat <1.1).

**Figure 1.** Correlation plot

**S1.** Assessment of serial measurements over time

|  | Number of patients/  Number of total repeated observations | Median number of serial measurements in total population* | Median number of serial measurements in males* (overall: 9(3,24)) | Median number of serial measurements in females* (overall: 6(2,18)) | p-value |
| --- | --- | --- | --- | --- | --- |
| Weight | 427/4880 | 4 (2,11) | 5 (2,12) | 4 (1,11) | 0.159 |
| BMI | 416/4849 | 5 (2,12) | 5 (2,12) | 4 (1,11) | 0.209 |
| Heart rate | 445/7097 | 5 (2,16) | 6 (2,16) | 5 (1,16) | 0.330 |
| Systolic blood pressure | 455/7402 | 5 (2,16) | 6 (2,16) | 5 (2,17) | 0.348 |
| Diastolic blood pressure | 454/7377 | 5 (2,16) | 6 (2,16) | 5 (2,16) | 0.286 |
| NT-proBNP | 394/1319 | 2 (1,3) | 2 (1,4) | 2 (1,3) | 0.335 |
| Hs-TnT | 209/646 | 2 (1,3) | 2 (1,4) | 2 (1,3) | 0.270 |
| CRP | 416/3094 | 3 (1,7) | 3 (1,9) | 2 (1,6) | 0.223 |
| CK | 126/356 | 2 (1,3) | 1 (1,2) | 2 (1,4) | 0.208 |
| CK-MB mass | 169/438 | 2 (1,3) | 2 (1,3) | 2 (1,3) | 0.925 |
| Albumin | 249/1927 | 3 (1,9) | 3 (1,10) | 3 (1,8) | 0.890 |
| Total protein | 119/497 | 2 (1,4) | 2 (1,5) | 2 (1,4) | 0.133 |
| Creatinine | 480/6157 | 5 (2,15) | 6 (2,15) | 4 (2,11) | **0.025** |
| Urea | 454/4196 | 3 (2,10) | 4 (2,10) | 3 (1,8) | 0.062 |
| CKD-EPI eGFR | 351/3899 | 4 (2,12) | 5 (2,12) | 4 (1,11) | 0.450 |
| Sodium | 469/5528 | 4 (2,12) | 6 (2,13) | 4 (2,11) | **0.020** |
| Potassium | 468/5595 | 4 (2,13) | 6 (2,13) | 4 (2,11) | **0.026** |
| Calcium | 2632/2113 | 4 (1,10) | 3 (2,10) | 4 (1,10) | 0.760 |
| AST | 307/3053 | 4 (1,11) | 4 (2,11) | 4 (2,10) | 0.348 |
| ALT | 315/3242 | 4 (2,12) | 4 (2,12) | 4 (1,12) | 0.361 |
| GGT | 300/2985 | 4 (1,11) | 4 (2,11) | 4 (1,10) | 0.176 |
| ALP | 290/2874 | 4 (1,11) | 4 (2,11) | 4 (1,10) | 0.587 |
| LDH | 295/2987 | 4 (1,11) | 5 (2,11) | 4 (1,10) | 0.338 |
| Total bilirubin | 278/2759 | 4 (1,11) | 3 (1,11) | 4 (1,9) | 0.702 |
| Total cholesterol | 163/413 | 2 (1,3) | 2 (1,3) | 2 (1,3) | 0.233 |
| HDL cholesterol | 159/389 | 2 (1,3) | 2 (1,3) | 2 (1,3) | 0.155 |
| LDL cholesterol | 231/524 | 2 (1,3) | 2 (1,3) | 1 (1,2) | **0.037** |
| Triglycerides | 148/385 | 2 (1,3) | 2 (1,3) | 1 (1,3) | 0.527 |
| Ferritin | 152/647 | 2 (1,4) | 2 (1,4) | 3 (2,4) | 0.135 |
| Iron | 144/440 | 2 (1,3) | 2 (1,4) | 2 (1,3) | 0.825 |
| Transferrin | 130/394 | 2 (1,3) | 2 (1,3) | 2 (1,3) | 0.925 |
| Haemoglobin | 468/5776 | 4(2,13) | 5 (2,14) | 4 (2,11) | **0.015** |
| Haematocrit | 309/2513 | 4 (2,9) | 4 (2,9) | 3 (1,8) | 0.127 |
| MCV | 468/5771 | 4 (2,13) | 5 (2,14) | 4 (2,11) | **0.015** |
| Leukocytes | 346/4972 | 6(2,16) | 6 (2,17) | 6 (2,15) | 0.292 |
| Thrombocytes | 350/4917 | 6 (2,15) | 6 (3,17) | 6 (2,14) | 0.237 |
| HbA1c | 124/367 | 2(1,4) | 2 (1,4) | 2 (1,4) | 0.556 |

*Values are presented as median (25^th^ and 75^th^ percentiles) for variables. Abbreviations are as defined previously. Table explains the available data of patients and their longitudinal total observation number for each variable.

**S2.** Single laboratory values analysis for all-cause mortality

|  | **Adjusted for age and sex** | | | **Adjusted for clinical variables^+^** | | |
| --- | --- | --- | --- | --- | --- | --- |
| **Variables** | **HR (95%CI)** | **P-value** | **AUC** | **HR (95%CI)** | **P-value** | **AUC** |
| NT-proBNP* | 1.76 (1.52 - 2.04) | <0.001 | 0.641 | 1.73 (1.49 - 2.02) | <0.001 | 0.705 |
| Hs-TnT* | 1.82 (1.31 - 2.55) | <0.001 | 0.611 | 1.68 (1.20 - 2.40) | 0.001 | 0.683 |
| CRP* | 3.02 (2.36 - 3.91) | <0.001 | 0.636 | 2.80 (2.20 - 3.64) | <0.001 | 0.663 |
| CK* | 0.71 (0.45 - 1.10) | 0.130 | 0.616 | 0.78 (0.50 - 1.19) | 0.262 | 0.705 |
| CK-MB mass* | 0.72 (0.35 - 1.37) | 0.320 | 0.607 | 0.83 (0.41 - 1.57) | 0.586 | 0.698 |
| Albumin | 0.83 (0.78 - 0.88) | <0.001 | 0.545 | 0.83 (0.78 - 0.87) | <0.001 | 0.642 |
| Total protein | 0.94 (0.90 - 0.98) | 0.001 | 0.552 | 0.94 (0.91 - 0.98) | 0.002 | 0.688 |
| Creatinine | 1.003 (1.001 - 1.004) | <0.001 | 0.590 | 1.003 (1.001 - 1.004) | <0.001 | 0.694 |
| Urea | 1.13 (1.10 - 1.17) | <0.001 | 0.616 | 1.13 (1.10 - 1.16) | <0.001 | 0.680 |
| CKD-EPI eGFR * | 0.48 (0.35 - 0.69) | <0.001 | 0.570 | 0.50 (0.36 - 0.71) | <0.001 | 0.688 |
| Sodium | 0.85 (0.79 - 0.91) | <0.001 | 0.611 | 0.87 (0.81 - 0.93) | <0.001 | 0.684 |
| Potassium | 0.74 (0.37 - 1.50) | 0.401 | 0.596 | 0.66 (0.32 - 1.32) | 0.240 | 0.699 |
| Calcium | 0.53 (0.30 - 0.92) | 0.023 | 0.576 | 0.54 (0.325 - 0.91) | 0.023 | 0.694 |
| AST | 1.005 (1.002 - 1.007) | 0.002 | 0.590 | 1.005 (1.003 - 1.007) | <0.001 | 0.698 |
| ALT | 1.004 (1.002 - 1.007) | 0.004 | 0.589 | 1.005 (1.002 - 1.007) | 0.001 | 0.700 |
| GGT* | 1.95(1.54 - 2.50) | <0.001 | 0.659 | 1.91 (1.50 - 2.41) | <0.001 | 0.724 |
| ALP | 1.008 (1.005 - 1.010) | <0.001 | 0.640 | 1.008 (1.005 - 1.010) | <0.001 | 0.708 |
| LDH | 1.005 (1.004 - 1.007) | <0.001 | 0.544 | 1.005 (1.003 - 1.007) | <0.001 | 0.665 |
| Total bilirubin | 1.02 (1.01 - 1.03) | 0.009 | 0.582 | 1.02 (1.01 - 1.03) | 0.003 | 0.684 |
| Total cholesterol | 0.77 (0.52 - 1.10) | 0.163 | 0.618 | 0.90 (0.62 - 1.26) | 0.579 | 0.704 |
| HDL cholesterol* | 0.35 (0.05 - 2.42) | 0.285 | 0.573 | 0.53 (0.08 - 3.36) | 0.503 | 0.702 |
| LDL cholesterol | 0.73 (0.51 - 1.03) | 0.075 | 0.622 | 0.82 (0.57 - 1.13) | 0.240 | 0.710 |
| Triglycerides | 0.83 (0.42 - 1.48) | 0.579 | 0.588 | 0.88 (0.47 - 1.60) | 0.670 | 0.700 |
| Ferritin * | 1.64 (1.25 - 2.16) | <0.001 | 0.623 | 1.59 (1.23 - 2.06) | <0.001 | 0.697 |
| Iron* | 0.29 (0.10 - 0.80) | 0.017 | 0.575 | 0.30 (0.10 - 0.80) | 0.016 | 0.696 |
| Transferrin | 0.23 (0.09 - 0.54) | <0.001 | 0.582 | 0.28 (0.12 - 0.59) | 0.001 | 0.688 |
| Haemoglobin | 0.47 (0.38 - 0.57) | <0.001 | 0.565 | 0.50 (0.41 - 0.61) | <0.001 | 0.631 |
| Haematocrit | 0.87 (0.83 - 0.92) | <0.001 | 0.543 | 0.89 (0.85 - 0.93) | <0.001 | 0.636 |
| MCV | 1.01 (0.99 – 1.04) | 0.318 | 0.593 | 1.01 (0.99 - 1.04) | 0.301 | 0.704 |
| Leukocytes | 1.09 (1.04 - 1.14) | <0.001 | 0.598 | 1.09 (1.04 - 1.14) | <0.001 | 0.692 |
| Thrombocytes * | 0.45 (0.31 - 0.67) | <0.001 | 0.590 | 0.43 (0.30 - 0.63) | <0.001 | 0.707 |
| HbA1c | 0.98 (0.94 - 1.02) | 0.330 | 0.576 | 0.98 (0.94 - 1.01) | 0.192 | 0.699 |

*Values are log-transformed.

**^+^** Model for clinical variables was adjusted for age, sex, heart rate, body mass index (BMI), and diastolic blood pressure.

**BMI:** Body mass index; **NT-proBNP:** N-terminal prohormone of brain natriuretic peptide; **Hs-TnT:** High-sensitivity troponin;

**CRP:** C reactive protein; **CK:** Creatine kinase; **CK-MB mass:** Creatine kinase – MB mass;

**CKD-EPI eGFR:** Glomerular Filtration Rate obtained from CKD-EPI equations; **AST:** Aspartate aminotransferase;

**ALT:** Alanine aminotransferase; **GGT:** Gamma-glutamyl transferase; **ALP:** Alkaline phosphatase; **LDH:** Lactase dehydrogenase;

**HDL cholesterol:** High-density lipoprotein; **LDL cholesterol:** Low-density lipoprotein; **MCV:** Mean corpuscular volume.

This figure displays the hazard ratios (HRs) and 95% credible intervals for each laboratory values analyzed in univariate joint models.

**S3.** Single laboratory values analysis for all-cause mortality adjusted for time-varying measurements

|  | **Adjusted for baseline clinical variables^+^** | | | **Adjusted for baseline clinical covariates and time-varying measurements** | | |
| --- | --- | --- | --- | --- | --- | --- |
| **Variables** | **HR (95% CI)** | **P-value** | **AUC** | **HR (95%CI)** | **P-value** | **AUC** |
| NT-proBNP* | 1.73 (1.49 - 2.02) | <0.001 | 0.705 | 1.77 (1.51 - 2.08) | <0.001 | 0.693 |
| Hs-TnT* | 1.68 (1.20 - 2.40) | 0.001 | 0.683 | 1.73 (1.25 - 2.43) | <0.001 | 0.678 |
| CRP* | 2.80 (2.20 - 3.64) | <0.001 | 0.663 | 2.63 (2.05 - 3.51) | <0.001 | 0.649 |
| CK* | 0.78 (0.50 - 1.19) | 0.262 | 0.705 | 0.73 (0.46 - 1.12) | 0.152 | 0.697 |
| CK-MB mass* | 0.83 (0.41 - 1.57) | 0.586 | 0.698 | 0.77 (0.38 - 1.45) | 0.456 | 0.694 |
| Albumin | 0.83 (0.78 - 0.87) | <0.001 | 0.642 | 0.83 (0.78 - 0.88) | <0.001 | 0.618 |
| Total protein | 0.94 (0.91 - 0.98) | 0.002 | 0.688 | 0.94 (0.90 - 0.98) | <0.001 | 0.638 |
| Creatinine | 1.003 (1.001 - 1.004) | <0.001 | 0.694 | 1.002 (1.0002 - 1.003) | 0.032 | 0.674 |
| Urea | 1.13 (1.10 - 1.16) | <0.001 | 0.680 | 1.12 (1.09 - 1.15) | <0.001 | 0.687 |
| CKD-EPI eGFR * | 0.50 (0.36 - 0.71) | <0.001 | 0.688 | 0.72 (0.49 - 1.08) | 0.110 | 0.696 |
| Sodium | 0.87 (0.81 - 0.93) | <0.001 | 0.684 | 0.87 (0.81 - 0.93) | <0.001 | 0.649 |
| Potassium | 0.66 (0.32 - 1.32) | 0.240 | 0.699 | 0.67 (0.34 - 1.32) | 0.247 | 0.668 |
| Calcium | 0.54 (0.325 - 0.91) | 0.023 | 0.694 | 0.54 (0.33 - 0.89) | 0.016 | 0.683 |
| AST | 1.005 (1.003 - 1.007) | <0.001 | 0.698 | 1.005 (1.003 - 1.007) | <0.001 | 0.678 |
| ALT | 1.005 (1.002 - 1.007) | 0.001 | 0.700 | 1.005 (1.003 - 1.007) | <0.001 | 0.679 |
| GGT* | 1.91 (1.50 - 2.41) | <0.001 | 0.724 | 1.71 (1.33 - 2.19) | <0.001 | 0.722 |
| ALP | 1.008 (1.005 - 1.010) | <0.001 | 0.708 | 1.007 (1.004 - 1.010) | <0.001 | 0.669 |
| LDH | 1.005 (1.003 - 1.007) | <0.001 | 0.665 | 1.005 (1.004 - 1.007) | <0.001 | 0.670 |
| Total bilirubin | 1.02 (1.01 - 1.03) | 0.003 | 0.684 | 1.02 (1.01 - 1.03) | 0.002 | 0.683 |
| Total cholesterol | 0.90 (0.62 - 1.26) | 0.579 | 0.704 | 0.90 (0.61 - 1.28) | 0.570 | 0.692 |
| HDL cholesterol* | 0.53 (0.08 - 3.36) | 0.503 | 0.702 | 0.48 (0.06 - 3.86) | 0.464 | 0.702 |
| LDL cholesterol | 0.82 (0.57 - 1.13) | 0.240 | 0.710 | 0.83 (0.58 - 1.162) | 0.300 | 0.704 |
| Triglycerides | 0.88 (0.47 - 1.60) | 0.670 | 0.700 | 0.83 (0.41 - 1.54) | 0.590 | 0.684 |
| Ferritin * | 1.59 (1.23 - 2.06) | <0.001 | 0.697 | 1.59 (1.23 - 2.09) | <0.001 | 0.690 |
| Iron* | 0.30 (0.10 - 0.80) | 0.016 | 0.696 | 0.24 (0.05 - 0.74) | 0.009 | 0.681 |
| Transferrin | 0.28 (0.12 - 0.59) | 0.001 | 0.688 | 0.29 (0.11 - 0.64) | 0.001 | 0.682 |
| Haemoglobin | 0.50 (0.41 - 0.61) | <0.001 | 0.631 | 0.56 (0.45 - 0.70) | <0.001 | 0.639 |
| Haematocrit | 0.89 (0.85 - 0.93) | <0.001 | 0.636 | 0.89 (0.85 - 0.94) | <0.001 | 0.631 |
| MCV | 1.01 (0.99 - 1.04) | 0.301 | 0.704 | 1.004 (0.98 - 1.03) | 0.786 | 0.690 |
| Leukocytes | 1.09 (1.04 - 1.14) | <0.001 | 0.692 | 1.06 (1.02 - 1.10) | 0.006 | 0.671 |
| Thrombocytes * | 0.43 (0.30 - 0.63) | <0.001 | 0.707 | 0.56 (0.39 - 0.85) | 0.006 | 0.689 |
| HbA1c | 0.98 (0.94 - 1.01) | 0.192 | 0.699 | 0.98 (0.94 - 1.01) | 0.224 | 0.667 |

*Values are log-transformed.

**^+^** Model adjusted for clinical variables was adjusted for age, sex, heart rate, body mass index (BMI), and diastolic blood pressure.

**BMI:** Body mass index; **NT-proBNP:** N-terminal prohormone of brain natriuretic peptide; **Hs-TnT:** High-sensitivity troponin;

**CRP:** C reactive protein; **CK:** Creatine kinase; **CK-MB mass:** Creatine kinase – MB mass;

**CKD-EPI eGFR:** Glomerular Filtration Rate obtained from CKD-EPI equations; **AST:** Aspartate aminotransferase;

**ALT:** Alanine aminotransferase; **GGT:** Gamma-glutamyl transferase; **ALP:** Alkaline phosphatase; **LDH:** Lactase dehydrogenase;

**HDL cholesterol:** High-density lipoprotein; **LDL cholesterol:** Low-density lipoprotein; **MCV:** Mean corpuscular volume.

This table displays the hazard ratios (HRs) and 95% credible intervals for each of the laboratory values analyzed in univariate joint models.

**S4.** Sex-based comparison of associations of serially measured single laboratory values with all-cause mortality, adjusted for age

|  | Males | | Females | |  |
| --- | --- | --- | --- | --- | --- |
| **Variables** | **HR (95%CI)** | **P-value** | **HR (95%CI)** | **P-value** | **p-interaction** |
| NT-proBNP* | 1.94(1.50-2.50) | <0.001 | 1.66(1.39-1.99) | <0.001 | 0.357 |
| Hs-TnT* | 2.36(1.39-4.12) | 0.002 | 1.63(1.10-2.41) | 0.015 | 0.213 |
| CRP* | 3.01(2.19-4.12) | <0.001 | 3.06(2.13-4.56) | <0.001 | 0.944 |
| CK* | 0.63(0.26-1.28) | 0.228 | 0.74(0.43-1.21) | 0.234 | 0.735 |
| CK-MB mass* | 1.01(0.28 -2.67) | 0.909 | 0.63(0.25-1.44) | 0.283 | 0.438 |
| Albumin | 0.86(0.79-0.92) | <0.001 | 0.80(0.75-0.87) | <0.001 | 0.149 |
| Total protein | 0.96(0.90-1.01) | 0.133 | 0.92(0.88-0.96) | 0.002 | 0.236 |
| Creatinine | 1.005(1.002-1.007) | 0.006 | 1.002(1.001-1.004) | 0.015 | 0.147 |
| Urea | 1.12(1.07-1.66) | <0.001 | 1.15(1.10-1.19) | <0.001 | 0.395 |
| CKD-EPI eGFR* | 0.51(0.29-0.95) | 0.036 | 0.46(0.31-0.71) | 0.002 | 0.809 |
| Sodium | 0.84(0.78-0.90) | <0.001 | 0.85(0.79-0.91) | <0.001 | 0.431 |
| Potassium | 0.59(0.23-1.39) | 0.245 | 0.90(0.39-2.04) | 0.806 | 0.434 |
| Calcium | 0.52(0.25-1.01) | 0.053 | 0.55(0.30-1.02) | 0.734 | 0.936 |
| AST | 1.005(1.002-1.008) | 0.012 | 1.004(1.0001-1.007) | 0.046 | 0.486 |
| ALT | 1.011(1.005-1.015) | 0.008 | 1.003(0.99-1.006) | 0.068 | **0.031** |
| GGT* | 1.90(1.41-2.57) | <0.001 | 2.01(1.35-3.04) | 0.002 | 0.838 |
| ALP | 1.007(1.004-1.010) | <0.001 | 1.011(1.004-1.019) | 0.003 | 0.292 |
| LDH | 1.006(1.003-1.010) | 0.001 | 1.005(1.004-1.007) | <0.001 | 0.864 |
| Total bilirubin | 1.02(1.01-1.03) | <0.001 | 0.99(0.95-1.02) | 0.471 | **0.022** |
| Total cholesterol | 0.81(0.23-1.59) | 0.668 | 0.76(0.48-1.14) | 0.206 | 0.705 |
| HDL cholesterol* | 1.51(0.22-13.76) | 0.738 | 0.02(0.004-0.33) | 0.007 | **0.004** |
| LDL cholesterol | 1.23(0.70-2.00) | 0.405 | 0.60(0.38-0.91) | 0.011 | **0.042** |
| Triglycerides | 0.16(0.04-0.74) | 0.014 | 1.09(0.56-2.03) | 0.783 | **0.008** |
| Ferritin* | 1.83(1.19-2.91) | 0.009 | 1.56(1.15-2.13) | 0.004 | 0.520 |
| Iron* | 0.61(0.14-2.19) | 0.462 | 0.16(0.04-0.61) | 0.005 | 0.094 |
| Transferrin | 0.26(0.07-0.88) | 0.025 | 0.18(0.06-0.51) | <0.001 | 0.680 |
| Haemoglobin | 0.47(0.36-0.58) | <0.001 | 0.48(0.34-0.65) | <0.001 | 0.981 |
| Haematocrit | 0.86(0.80-0.92) | <0.001 | 0.90(0.84-0.96) | 0.001 | 0.312 |
| MCV | 1.02(0.99-1.05) | 0.166 | 0.99(0.96-1.04) | 0.946 | 0.313 |
| Leukocytes | 1.10(0.96-1.24) | 0.190 | 1.17(1.08-1.25) | <0.001 | 0.341 |
| Thrombocytes* | 0.34(0.22-0.55) | <0.001 | 0.64(0.39-1.13) | 0.117 | 0.070 |
| HbA1c | 0.94(0.88-1.03) | 0.177 | 0.99(0.96-1.03) | 0.828 | 0.235 |

*Values are log-transformed.

**BMI:** Body mass index; **NT-proBNP:** N-terminal prohormone of brain natriuretic peptide; **Hs-TnT:** High-sensitivity troponin;

**CRP:** C reactive protein; **CK:** Creatine kinase; **CK-MB mass:** Creatine kinase – MB mass;

**CKD-EPI eGFR:** Glomerular Filtration Rate obtained from CKD-EPI equations; **AST:** Aspartate aminotransferase;

**ALT:** Alanine aminotransferase; **GGT:** Gamma-glutamyl transferase; **ALP:** Alkaline phosphatase; **LDH:** Lactase dehydrogenase;

**HDL cholesterol:** High-density lipoprotein; **LDL cholesterol:** Low-density lipoprotein; **MCV:** Mean corpuscular volume..

This figure displays the hazard ratios (HRs) and 95% credible intervals for each laboratory values analyzed in univariate joint models. Models were adjusted for age.

**S5.** Sex-based comparison of associations of serially measured single laboratory values with all-cause mortality, adjusted for clinical characteristics

|  | **Males** | | **Females** | |  |
| --- | --- | --- | --- | --- | --- |
| **Variables** | **HR (95%CI)** | **p-value** | **HR (95%CI)** | **p-value** | **p-interaction** |
| NT-proBNP* | 1.89 (1.46-2.46) | <0.001 | 1.66 (1.38-2.00) | <0.001 | 0.470 |
| Hs-TnT* | 2.24 (1.34 - 3.84) | 0.002 | 1.44 (0.99 - 2.12) | 0.053 | 0.140 |
| CRP* | 2.89 (2.12 - 4.32) | <0.001 | 2.80 (1.94 - 4.12) | <0.001 | 0.955 |
| CK* | 0.73 (0.36 - 1.46) | 0.365 | 0.77 (0.45 - 1.23) | 0.293 | 0.804 |
| CK-MB mass* | 1.15 (0.28 - 4.02) | 0.798 | 0.67 (0.28 - 1.51) | 0.344 | 0.498 |
| Albumin | 0.85 (0.79 - 0.91) | <0.001 | 0.80 (0.75 - 0.86) | <0.001 | 0.154 |
| Total protein | 0.97 (0.92 - 1.01) | 0.155 | 0.93 (0.89 - 0.97) | <0.001 | 0.107 |
| Creatinine | 1.005 (1.002 - 1.008) | 0.002 | 1.002 (1.001 - 1.004) | 0.017 | 0.130 |
| Urea | 1.12 (1.07 - 1.17) | <0.001 | 1.14 (1.10 - 1.18) | <0.001 | 0.450 |
| CKD-EPI eGFR* | 0.56 (0.32 - 0.97) | 0.040 | 0.50 (0.35 - 0.75) | 0.001 | 0.746 |
| Sodium | 0.86 (0.80 - 0.93) | <0.001 | 0.87 (0.81 - 0.93) | <0.001 | 0.463 |
| Potassium | 0.53 (0.22 - 1.22) | 0.137 | 0.77 (0.33 - 1.81) | 0.555 | 0.356 |
| Calcium | 0.49 (0.26 - 0.91) | 0.024 | 0.56 (0.33 - 0.98) | 0.044 | 0.579 |
| AST | 1.006 (1.002 - 1.009) | 0.009 | 1.004 (1.001 - 1.007) | 0.047 | 0.390 |
| ALT^#^ | 1.01 (1.006 - 1.017) | 0.001 | 1.004 (1.001 - 1.006) | 0.032 | **0.024** |
| GGT* | 1.91 (1.40 - 2.56) | <0.001 | 1.94 (1.29 - 2.92) | 0.002 | 0.948 |
| ALP | 1.008 (1.004 - 1.01) | <0.001 | 1.011 (1.004 - 1.02) | 0.001 | 0.291 |
| LDH | 1.006 (1.002 - 1.010) | 0.001 | 1.005 (1.003 - 1.007) | <0.001 | 0.706 |
| Total bilirubin^#^ | 1.03 (1.02 - 1.04) | <0.001 | 0.99 (0.96 - 1.02) | 0.553 | **0.020** |
| Total cholesterol | 0.90 (0.43 - 1.67) | 0.776 | 0.87 (0.57 - 1.27) | 0.492 | 0.950 |
| HDL cholesterol*^#^ | 2.00 (0.18 - 27.25) | 0.596 | 0.04 (0.001 - 0.78) | 0.034 | **0.004** |
| LDL cholesterol^#^ | 1.35 (0.75 - 2.19) | 0.258 | 0.66 (0.42 - 0.99) | 0.043 | **0.040** |
| Triglycerides^#^ | 0.18 (0.03 - 0.66) | 0.010 | 1.22 (0.59 - 2.38) | 0.546 | **0.008** |
| Ferritin* | 1.83 (1.19 - 2.83) | 0.003 | 1.51 (1.14 - 2.02) | 0.005 | 0.471 |
| Iron* | 0.58 (0.14 - 2.09) | 0.400 | 0.15 (0.03 - 0.56) | 0.005 | 0.065 |
| Transferrin | 0.31 (0.09 - 1.03) | 0.056 | 0.24 (0.07 - 0.60) | 0.001 | 0.790 |
| Haemoglobin | 0.50 (0.40 - 0.63) | <0.001 | 0.50 (0.37 - 0.67) | <0.001 | 0.934 |
| Haematocrit | 0.87 (0.82 - 0.92) | <0.001 | 0.91 (0.85 - 0.96) | 0.002 | 0.308 |
| MCV | 1.02 (0.99-1.05) | 0.172 | 1.004 (0.97 – 1.04) | 0.831 | 0.360 |
| Leukocytes | 1.09 (0.98 - 1.24) | 0.124 | 1.16 (1.08 - 1.24) | <0.001 | 0.373 |
| Thrombocytes* | 0.35 (0.23 - 0.55) | <0.001 | 0.55 (0.33 - 0.93) | 0.029 | 0.104 |
| HbA1c | 0.94 (0.87 - 1.02) | 0.129 | 0.98 (0.94 - 1.02) | 0.285 | 0.308 |

*Values are log-transformed. ^#^Laboratory values also additionally adjusted for time varying measurements, and their respective adjusted p values for interaction terms are: ALT p-interaction: 0.026, total bilirubin p-interaction: 0.019, HDL cholesterol p-interaction: 0.007; LDL cholesterol p-interaction: 0.042; Triglyceride p-interaction: 0.003.

**NT-proBNP:** N-terminal prohormone of brain natriuretic peptide; **Hs-TnT:** High-sensitivity troponin;

**CRP:** C reactive protein; **CK:** Creatine kinase; **CK-MB mass:** Creatine kinase – MB mass;

**CKD-EPI eGFR:** Glomerular Filtration Rate obtained from CKD-EPI equations; **AST:** Aspartate aminotransferase;

**ALT:** Alanine aminotransferase; **GGT:** Gamma-glutamyl transferase; **ALP:** Alkaline phosphatase; **LDH:** Lactase dehydrogenase;

**HDL cholesterol:** High-density lipoprotein; **LDL cholesterol:** Low-density lipoprotein; **MCV:** Mean corpuscular volume.

This figure displays the hazard ratios (HRs) and 95% credible intervals for single laboratory values joint models. Models were adjusted for age, heart rate, body mass index (BMI), and diastolic blood pressure.

**S6.** Multiple laboratory values models: associations of serially measured laboratory values with all-cause mortality

|  | **Adjusted for age and sex** | | |  |
| --- | --- | --- | --- | --- |
| **Model** | **Variables** | **HR (95%CI)** | **P-value** | **AUC** |
| Model 1 | NT-proBNP* | 1.35(1.06-1.74) | 0.015 | 0.657 |
|  | Hs-TnT * | 1.15(0.70-1.82) | 0.570 |  |
|  | CRP* | 2.55(1.98-3.35) | <0.001 |  |
| Model 2 | NT-proBNP* | 1.75 (1.44 - 2.14) | <0.001 | 0.651 |
|  | Albumin | 0.86 (0.82 - 0.90) | <0.001 |  |
|  | AST | 1.003 (1.00 - 1.006) | 0.051 |  |
|  | GGT* | 1.92 (1.32 - 2.80) | 0.011 |  |
|  | ALP | 0.998 (0.993 - 1.004) | 0.589 |  |
| Model 3 | NT-proBNP* | 2.05 (1.59 - 2.71) | <0.001 | 0.649 |
|  | CKD-EPI eGFR | 2.45 (1.36 - 4.58) | 0.002 |  |
|  | Albumin | 0.82 (0.76 - 0.88) | <0.001 |  |
|  | Sodium | 0.93 (0.85 - 1.02) | 0.129 |  |
|  | Calcium | 1.42 (0.78 - 2.64) | 0.247 |  |
| Model 4 | NT-proBNP* | 1.37 (1.07 - 1.63) | 0.011 | 0.680 |
|  | CRP* | 2.35 (1.78 - 3.22) | <0.001 |  |
|  | Ferritin* | 1.09 (0.75 - 1.58) | 0.643 |  |
|  | LDH | 1.004 (1.002 - 1.006) | <0.001 |  |
|  | Leukocytes | 1.02 (0.95 - 1.07) | 0.564 |  |
| Model 5 | NT-proBNP* | 1.44 (1.19 - 1.76) | <0.001 | 0.611 |
|  | Ferritin* | 1.17 (0.83 - 1.64) | 0.381 |  |
|  | Hemoglobin | 0.60 (0.48 - 0.75) | <0.001 |  |
|  | MCV | 1.002 (0.97 - 1.03) | 0.872 |  |

*Values are log-transformed.

**NT-proBNP:** N-terminal prohormone of brain natriuretic peptide; **Hs-TnT:** High-sensitivity troponin;

**CRP:** C reactive protein

**CKD-EPI eGFR:** Glomerular Filtration Rate obtained from CKD-EPI equations; **AST:** Aspartate aminotransferase;

**ALT:** Alanine aminotransferase; **GGT:** Gamma-glutamyl transferase; **ALP:** Alkaline phosphatase; **LDH:** Lactase dehydrogenase; **MCV:** Mean corpuscular volume.

This figure displays the hazard ratios (HRs) and 95% credible intervals for each laboratory values analyzed in multivariate joint models. Models were adjusted for age and sex.

**S7.** Landmark analysis of single and multiple laboratory value models

|  | **2-years (Main analysis)** | | | **1-year** | | | **6-months** | | |
| --- | --- | --- | --- | --- | --- | --- | --- | --- | --- |
| **Variables** | **AUC** | **Brier Score** | **ICI** | **AUC** | **Brier Score** | **ICI** | **AUC** | **Brier Score** | **ICI** |
| NT-proBNP | 0.705 | 0.146 | 0.029 | 0.699 | 0.176 | 0.030 | 0.700 | 0.189 | 0.013 |
| Hs-TnT | 0.683 | 0.146 | 0.045 | 0.670 | 0.182 | 0.034 | 0.679 | 0.193 | 0.044 |
| CRP | 0.663 | 0.146 | 0.050 | 0.681 | 0.176 | 0.030 | 0.707 | 0.186 | 0.051 |
| CK | 0.705 | 0.146 | 0.041 | 0.673 | 0.183 | 0.032 | 0.682 | 0.195 | 0.034 |
| CK-MB mass | 0.698 | 0.146 | 0.039 | 0.674 | 0.183 | 0.034 | 0.681 | 0.195 | 0.035 |
| Albumin | 0.642 | 0.159 | 0.027 | 0.654 | 0.193 | 0.025 | 0.672 | 0.201 | 0.017 |
| Total protein | 0.688 | 0.150 | 0.023 | 0.662 | 0.188 | 0.014 | 0.678 | 0.198 | 0.022 |
| Creatinine | 0.694 | 0.147 | 0.040 | 0.677 | 0.183 | 0.042 | 0.690 | 0.193 | 0.043 |
| Urea | 0.680 | 0.149 | 0.034 | 0.682 | 0.178 | 0.030 | 0.687 | 0.191 | 0.033 |
| CKD-EPI eGFR | 0.688 | 0.148 | 0.034 | 0.678 | 0.183 | 0.039 | 0.680 | 0.195 | 0.041 |
| Sodium | 0.684 | 0.146 | 0.018 | 0.663 | 0.185 | 0.030 | 0.684 | 0.194 | 0.022 |
| Potassium | 0.699 | 0.145 | 0.036 | 0.677 | 0.183 | 0.037 | 0.683 | 0.194 | 0.040 |
| Calcium | 0.694 | 0.146 | 0.039 | 0.681 | 0.181 | 0.033 | 0.687 | 0.192 | 0.036 |
| AST | 0.698 | 0.146 | 0.029 | 0.672 | 0.184 | 0.027 | 0.684 | 0.195 | 0.029 |
| ALT | 0.700 | 0.146 | 0.032 | 0.672 | 0.184 | 0.030 | 0.686 | 0.194 | 0.027 |
| GGT | 0.724 | 0.141 | 0.044 | 0.690 | 0.180 | 0.031 | 0.710 | 0.189 | 0.042 |
| ALP | 0.708 | 0.148 | 0.049 | 0.674 | 0.184 | 0.036 | 0.686 | 0.194 | 0.036 |
| LDH | 0.665 | 0.155 | 0.030 | 0.656 | 0.191 | 0.023 | 0.668 | 0.202 | 0.020 |
| Total bilirubin | 0.684 | 0.149 | 0.028 | 0.661 | 0.186 | 0.016 | 0.670 | 0.197 | 0.018 |
| Total cholesterol | 0.704 | 0.146 | 0.048 | 0.674 | 0.183 | 0.041 | 0.682 | 0.195 | 0.043 |
| HDL cholesterol | 0.702 | 0.145 | 0.053 | 0.672 | 0.181 | 0.044 | 0.681 | 0.193 | 0.044 |
| LDL cholesterol | 0.710 | 0.146 | 0.044 | 0.673 | 0.185 | 0.034 | 0.685 | 0.196 | 0.041 |
| Triglycerides | 0.700 | 0.146 | 0.051 | 0.668 | 0.183 | 0.041 | 0.680 | 0.195 | 0.048 |
| Ferritin | 0.697 | 0.143 | 0.032 | 0.676 | 0.181 | 0.032 | 0.686 | 0.192 | 0.031 |
| Iron | 0.696 | 0.145 | 0.053 | 0.674 | 0.181 | 0.054 | 0.684 | 0.193 | 0.048 |
| Transferrin | 0.688 | 0.143 | 0.050 | 0.666 | 0.180 | 0.050 | 0.688 | 0.189 | 0.052 |
| Haemoglobin | 0.631 | 0.155 | 0.039 | 0.685 | 0.180 | 0.009 | 0.671 | 0.196 | 0.038 |
| Haematocrit | 0.636 | 0.153 | 0.023 | 0.665 | 0.186 | 0.013 | 0.672 | 0.196 | 0.022 |
| MCV | 0.704 | 0.146 | 0.034 | 0.678 | 0.184 | 0.033 | 0.689 | 0.195 | 0.034 |
| Leukocytes | 0.692 | 0.147 | 0.026 | 0.666 | 0.186 | 0.023 | 0.675 | 0.198 | 0.037 |
| Thrombocytes | 0.707 | 0.146 | 0.059 | 0.683 | 0.180 | 0.026 | 0.686 | 0.192 | 0.022 |
| HbA1c | 0.699 | 0.147 | 0.034 | 0.669 | 0.185 | 0.026 | 0.681 | 0.196 | 0.027 |
| **Multi models** | | | | | | | | | |
| Model 1 | 0.701 | 0.148 | 0.058 | 0.721 | 0.169 | 0.033 | 0.721 | 0.183 | 0.044 |
| Model 2 | 0.706 | 0.151 | 0.030 | 0.702 | 0.181 | 0.012 | 0.705 | 0.192 | 0.020 |
| Model 3 | 0.699 | 0.151 | 0.038 | 0.691 | 0.182 | 0.038 | 0.700 | 0.192 | 0.020 |
| Model 4 | 0.715 | 0.145 | 0.041 | 0.720 | 0.172 | 0.027 | 0.730 | 0.183 | 0.036 |
| Model 5 | 0.672 | 0.149 | 0.047 | 0.718 | 0.170 | 0.029 | 0.703 | 0.186 | 0.020 |

To evaluate the discriminatory ability of each single- or multiple-laboratory-value model using time-varying ROC

curves, information from data collected up to each landmark time (6 months, 1 year and 2 years) was used to predict

outcomes during the subsequent follow-up period until year four.

**NT-proBNP:** N-terminal prohormone of brain natriuretic peptide; **Hs-TnT:** High-sensitivity troponin;

**CRP:** C reactive protein

**CKD-EPI eGFR:** Glomerular Filtration Rate obtained from CKD-EPI equations; **AST:** Aspartate aminotransferase;

**ALT:** Alanine aminotransferase; **GGT:** Gamma-glutamyl transferase; **ALP:** Alkaline phosphatase; **LDH:** Lactase dehydrogenase; **MCV:** Mean corpuscular volume. Models were adjusted for age, heart rate, body mass index (BMI), and diastolic blood pressure.

**S8.** Calibration plots of single and multiple laboratory values

|  | **2-years**  **(Main analysis)** | **1-year** | **6-months** |
| --- | --- | --- | --- |
| **Variables** |  |  |  |
| NT-proBNP |  |  |  |
| Hs-TnT |  |  |  |
| CRP |  |  |  |
| CK |  |  |  |
| CK-MB mass |  |  |  |
| Albumin |  |  |  |
| Total protein |  |  |  |
| Creatinine |  |  |  |
| Urea |  |  |  |
| CKD-EPI eGFR |  |  |  |
| Sodium |  |  |  |
| Potassium |  |  |  |
| Calcium |  |  |  |
| AST |  |  |  |
| ALT |  |  |  |
| GGT |  |  |  |
| ALP |  |  |  |
| LDH |  |  |  |
| Total bilirubin |  |  |  |
| Total cholesterol |  |  |  |
| HDL cholesterol |  |  |  |
| LDL cholesterol |  |  |  |
| Triglycerides |  |  |  |
| Ferritin |  |  |  |
| Iron |  |  |  |
| Transferrin |  |  |  |
| Haemoglobin |  |  |  |
| Haematocrit |  |  |  |
| MCV |  |  |  |
| Leukocytes |  |  |  |
| Thrombocytes |  |  |  |
| HbA1c |  |  |  |
| **Multi models** | | | |
| Model 1 |  |  |  |
| Model 2 |  |  |  |
| Model 3 |  |  |  |
| Model 4 |  |  |  |
| Model 5 |  |  |  |

**S9.** Discrimination comparison between NT-proBNP and multi models

|  | **Mean Bootstrap AUC (95% Bootstrap CI)** | **Mean Delta AUC**  **(95% Delta CI)** | **Bootstrap SE** | **Z-score** | **p-value** |
| --- | --- | --- | --- | --- | --- |
| **NT-proBNP**  **Model 1** | 0.704 (0.638-0.776)  0.698 (0.631-0.767) | -0.006 (-0.025- 0.012) | 0.011 | -0.5128 | 0.608 |
| **NT-proBNP**  **Model 2** | 0.707 (0.638-0.776)  0.697 (0.620-0.768) | -0.011 (-0.051- 0.016) | 0.019 | -0.5676 | 0.570 |
| **NT-proBNP**  **Model 3** | 0.708 (0.638-0.776)  0.693 (0.610-0.760) | -0.015 (-0.042- 0.013) | 0.016 | -0.9333 | 0.351 |
| **NT-proBNP**  **Model 4** | 0.704 (0.638-0.776)  0.688 (0.616-0.751) | -0.016 (-0.060- 0.017) | 0.022 | -0.7226 | 0.470 |
| **NT-proBNP**  **Model 5** | 0.708 (0.638-0.776)  0.701 (0.622-0.764) | -0.007 (-0.041- 0.012) | 0.016 | -0.4068 | 0.684 |

Models were adjusted for age, heart rate, body mass index (BMI), and diastolic blood pressure. To evaluate the discriminatory ability of each (single or multiple laboratory value) model with time-varying ROC curves, information from data collected up to year 2 was used to predict outcomes during consecutive 2-year periods.

Model 1: markers that were previously extensively studied in the context of HF (serial NT-proBNP, High-sensitivity troponin (Hs-TnT), and C reactive protein (CRP)).

Model 2: NT-proBNP plus liver-related biomarkers (albumin, aspartate aminotransferase (AST), Alkaline phosphatase (ALP), and Gamma- glutamyl transferase (GGT))

Model 3: NT-proBNP plus kidney related biomarkers (albumin, eGFR, sodium, calcium).

Model 4: NT-proBNP plus inflammatory biomarkers (CRP, ferritin, lactase dehydrogenase (LDH) and leukocytes). Model 5: NT-proBNP plus anemia-related biomarkers (ferritin, hemoglobin, MCV).
